# Virtual tumor mapping and margin control with 3-D planning and navigation

**DOI:** 10.1515/iss-2021-0009

**Published:** 2024-04-25

**Authors:** David Steybe, Pit J. Voss, Marc C. Metzger, Rainer Schmelzeisen, Philipp Poxleitner

**Affiliations:** Department of Oral and Maxillofacial Surgery, 14879 Faculty of Medicine, Medical Center – University of Freiburg, Freiburg, Germany; 14879 Berta-Ottenstein-Programme for Clinician Scientists, Faculty of Medicine, University of Freiburg, Freiburg, Germany

**Keywords:** head and neck cancer, intraoperative navigation, margin control, postoperative radiotherapy, tumor mapping

## Abstract

Computer technology–based treatment approaches like intraoperative navigation and intensity-modulated radiation therapy have become important components of state of the art head and neck cancer treatment. Multidirectional exchange of virtual three-dimensional patient data via an interdisciplinary platform allows all medical specialists involved in the patients treatment to take full advantage of these technologies. This review article gives an overview of current technologies and future directions regarding treatment approaches that are based on a virtual, three-dimensional patient specific dataset: storage and exchange of spatial information acquired via intraoperative navigation allow for a highly precise frozen section procedure. In the postoperative setting, virtual reconstruction of the tumor resection surface provides the basis for improved radiation therapy planning and virtual reconstruction of the tumor with integration of molecular findings creates a valuable tool for postoperative treatment and follow-up. These refinements of established treatment components and novel approaches have the potential to make a major contribution to improving the outcome in head and neck cancer patients.

## Introduction

State-of-the-art treatment of head and neck cancer requires an interdisciplinary approach with the setup of multidisciplinary teams recommended by international guidelines [1]. In this context, precise communication between all specialties involved is paramount. However, current strategies of interdisciplinary data exchange are usually based on written and verbally transmitted information. This approach comes along with the risk of inaccuracies due to interference issues and does not allow for the three-dimensional storage and exchange of spatial patient-specific findings, which is a limitation of particular relevance in the anatomically complex region of the head and neck.

In the past, advances in the field of computer technology have provided the basis for treatment approaches like computer-assisted surgery (CAS) and intensity-modulated radiation therapy (IMRT), which have become important components of head and neck cancer treatment [2, 3]. However, interdisciplinary real-time exchange of virtual three-dimensional patient data in order to take full advantage of such technologies is still uncommon.

**Figure 1:**
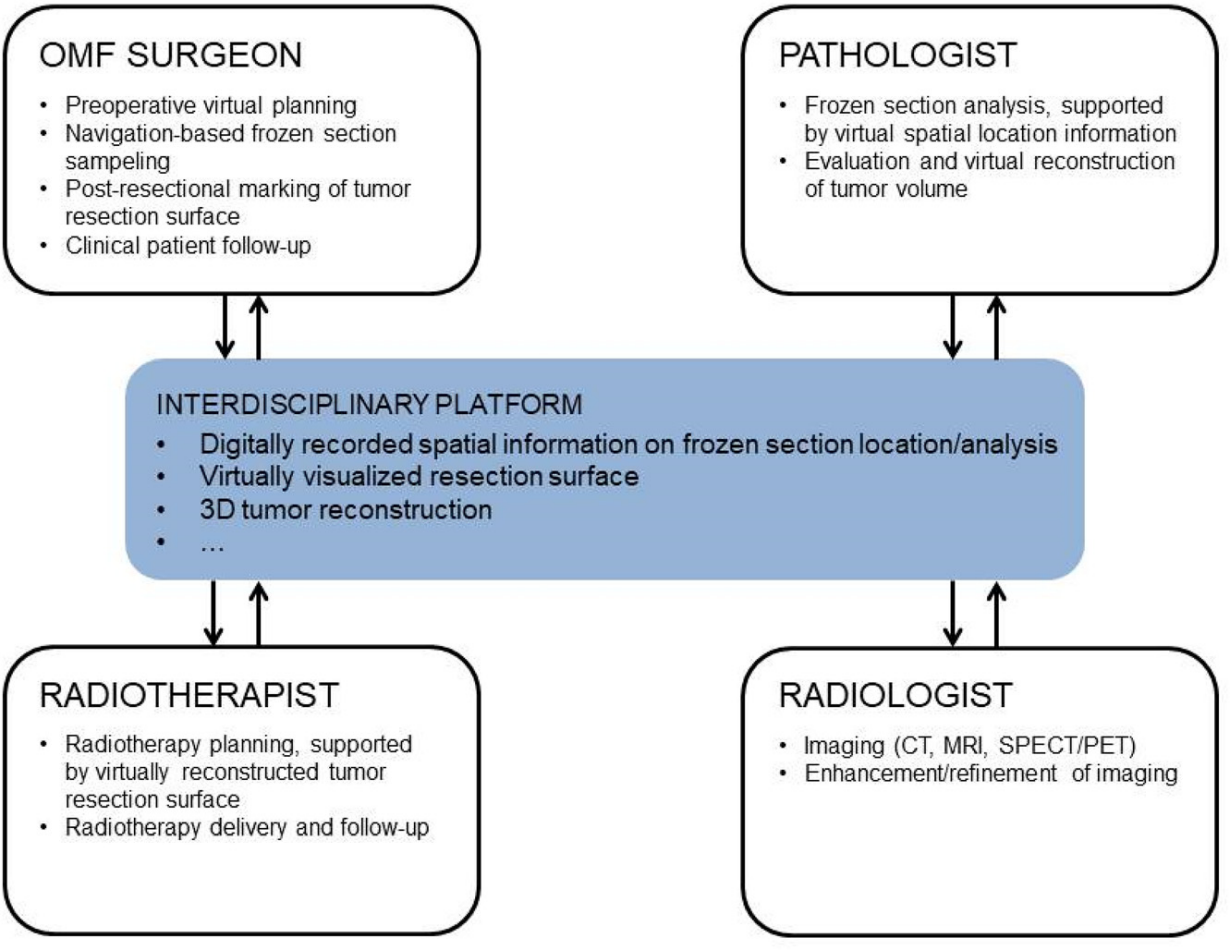
Scheme visualizing the platform-based multidirectional dataflow between medical specialties involved in head and neck cancer treatment.

A virtual network–based dataset of a patient can provide the basis for language-independent, precise storage and exchange of three-dimensionally visualized data between all medical specialists involved in diagnostic procedures, treatment planning, treatment delivery, and follow-up of a patient (Figure 1). The information included in the dataset can be accessed by each specialty involved at any time and be applied in all stages of the patient’s treatment. Once new data are generated during the course of therapy they can be added, making them available to all other specialties involved. This interdisciplinary, multidirectional dataflow enables standardization and increased precision of operative and postoperative therapy and thus has the potential to improve the outcome in surgical head and neck cancer treatment.

In this context, it is the aim of this article to give an overview of current technologies and future directions regarding approaches that can be integrated into a network-based, virtual, three-dimensional patient-specific dataset.

## Digitization of the frozen section analysis procedure

### Disciplines involved: oral and maxillofacial surgery, pathology

Among the factors determining the outcome in surgically treated head and neck cancer (ENT), the status of the resection margin is of utmost importance. The incidence of locoregional recurrence in OSCC patients with positive surgical margins has been reported to be up to twice that in those with negative margins [4], [5], [6]. However, the complexity and functional importance of the anatomical structures of the head and neck make it a challenging task to find a balance between complete resection and organ preservation in order to warrant good postoperative function and quality of life. Thus, intraoperative frozen section analysis provides a valuable tool to ensure complete surgical removal of the tumor while keeping the amount of resected tissue as low as possible.

In frozen section procedures, it is the current standard for the treating surgeon to orient the resected samples by using an individual, descriptive nomenclature, which is based on anatomical landmarks. This approach is not standardized, difficult to reproduce, and prone to errors [7], [8], [9]. In case of frozen sections determined to be tumor positive by pathohistological analysis, precise reorientation in the surgical field is crucial to locate and resect the remaining tumor. However, it has been questioned if the current standard approach, as described above, can provide the amount of accuracy required [10].

Within the last decade(s), computer-assisted surgery (CAS) has gained increasing relevance for different procedures in the field of oral and maxillofacial surgery, including cancer surgery [2, 11], [12], [13], [14], [15], [16]. In this context, intraoperative navigation provides the possibility to transfer a digital preoperative plan, including the segmented tumor volume and segmented vital structures (vessels, nerves), into the surgical field, thus allowing for guided tumor resection [17].

**Figure 2:**
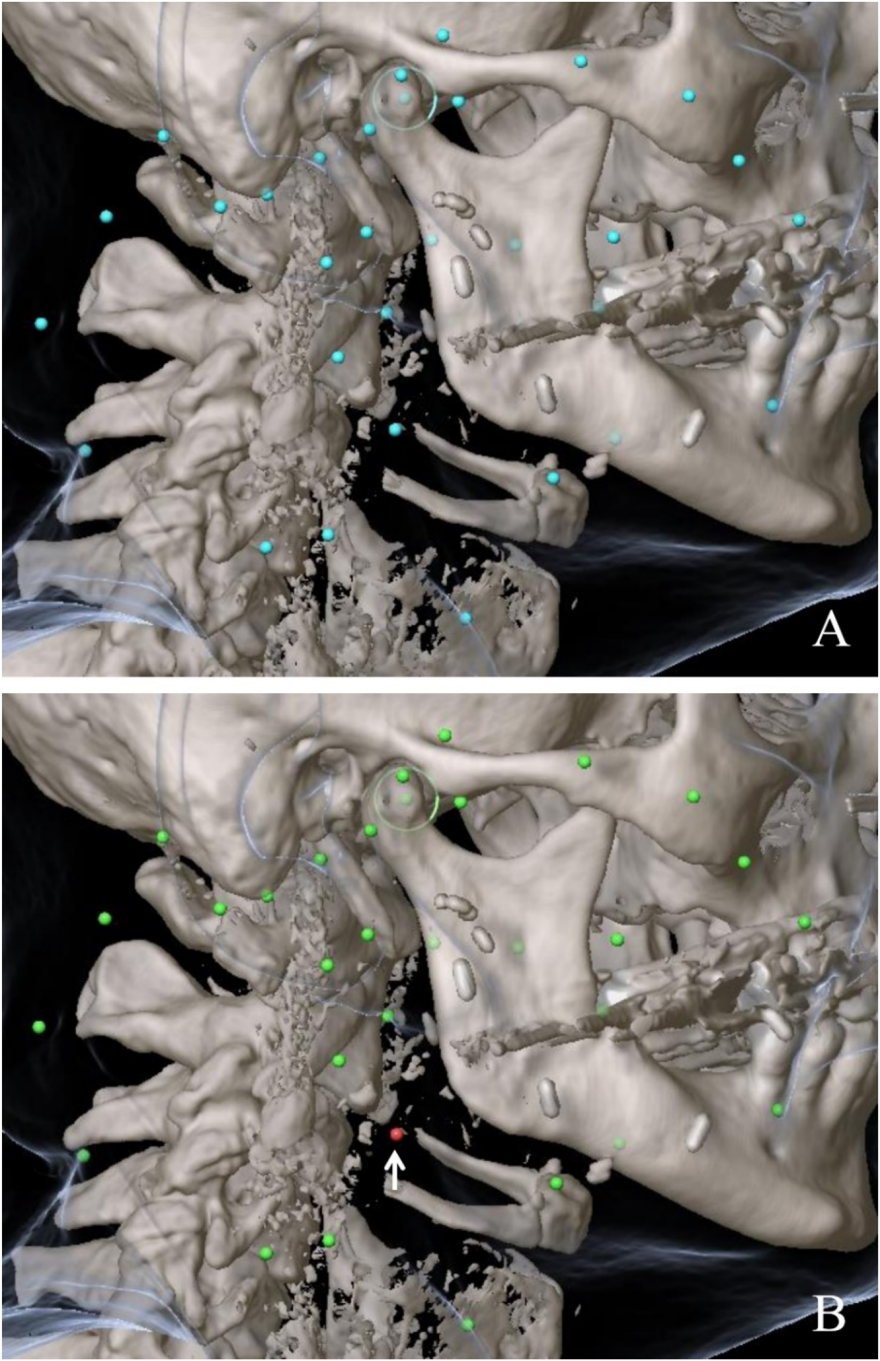
Navigation-based mapping during frozen section sampling. (A) Spatial coordinates for each sample (blue points) craeted in the patient’s virtual treatment plan. (B) Visualization of results of histopathological analysis of each sample by a standardized color code with arrow pointing at tumor positive section.

However, intraoperative navigation/computer-assisted surgery does not only allow for the transmission of virtual data into the surgical field but also for the incorporation of intraoperative information into a patient’s virtual dataset. This way, it can serve as a precise, language-independent digital communication tool at the interface between surgeon and pathologist in the context of the frozen section procedure [7, 8, 18, 19]: Following intraoperative navigation registration, pointers and surgical instruments can be displayed in the patient’s dataset in real time. When collecting frozen sections, the *in situ* location of every biopsy taken can be marked using the navigation probe, thus storing precise spatial coordinates for each sample in the patient’s virtual treatment plan. The labeled samples are transmitted to the pathologist who, via an interdisciplinary network-based platform, has remote access to the corresponding spatial information stored in the patient’s treatment plan; this way, the pathologist is able to precisely locate and orient each sample [19]. A standardized color code (green: negative resection margin, yellow: close resection margin, red: positive resection margin) is used by the pathologist to integrate the results of pathohistological analysis of each sample into the patient’s dataset (Figure 2). Having real-time access to this information via an interdisciplinary server enables the surgeon to perform navigation-based targeted re-resection in case of remaining cancer [7, 19]. In a clinical feasibility study, it has been demonstrated that intraoperative navigation is a viable method to facilitate anatomical definition of critical margins and positive frozen sections, enabling increased rates of tumor-free resection margins [18].

While intraoperative navigation/computer-aided surgery can provide high accuracy in relocating frozen section samples, a “defect-driven” approach has some inevitable limitations by itself: Applying this approach, with fragments of tissue from the surgical bed sent for pathohistological analysis, it is not possible for the pathologist to assess the distance between the surgical margin and the tumor cells. Moreover, it may be difficult for the pathologist to identify small clusters of tumor cells in the specimens, as correct information on the *in situ* position of the tumor bulk and thus on the spatial relation between the main specimen and the frozen section samples is missing [10].

Considering these aspects, some authors have opted for a “specimen-driven” approach to be preferred in frozen section procedures. In this approach, tissue is sampled directly from the resected specimen, allowing for the distance between the invasive front of the tumor and the resected front to be accurately assessed [10, 20].

Combining intraoperative navigation with 3D-scanning of the tumor specimen might provide a useful tool to establish a digitized, specimen-based frozen section procedure workflow. During the past years, intraoral scanning has gained increasing popularity in various fields of dentistry, including prosthodontics, implant dentistry, and orthodontics [21], [22], [23], [24]. Besides of being an accurate digital dental impression tool [25, 26], it has been demonstrated that acquiring 3D scans of intraoral soft tissue structures like the palate or edentulous jaws is possible using intraoral scanners as well [27], [28], [29].

**Figure 3:**
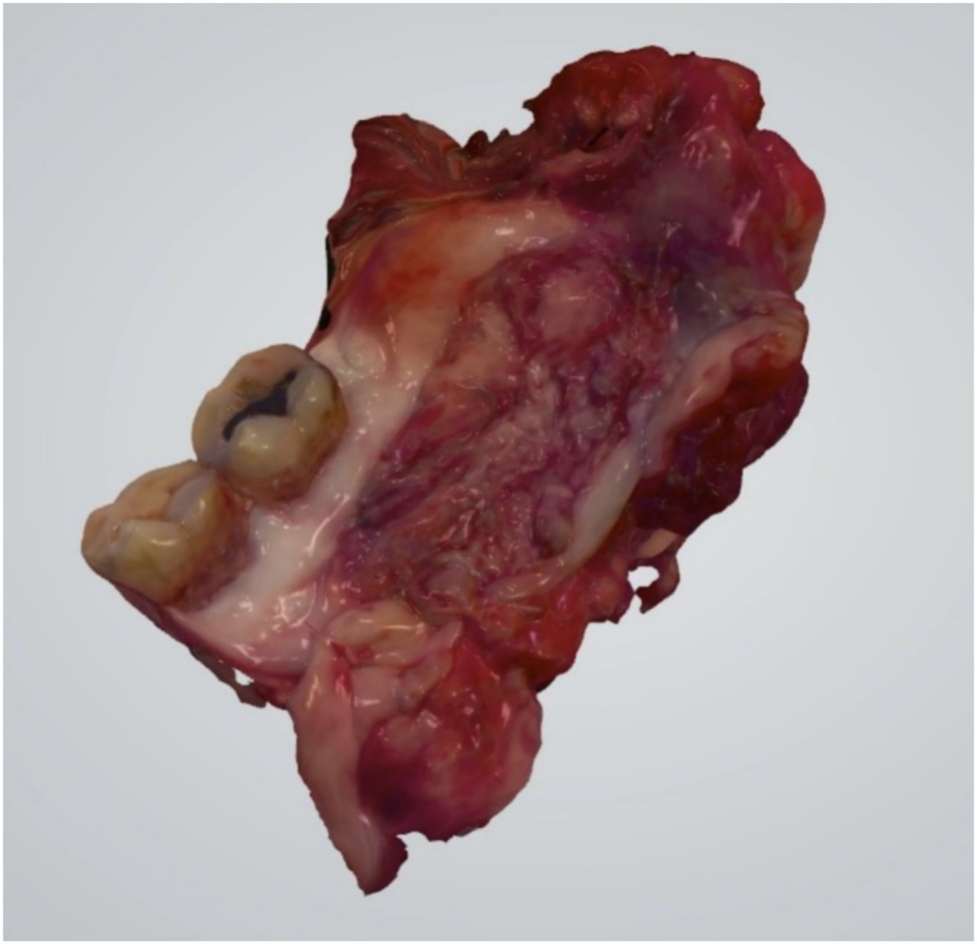
3D scan of the tumor resection specimen (acquired with intraoral scanner) as basis for a digitized, specimen-based frozen section procedure.

Taking this approach one step further, the authors could demonstrate that it is even possible to acquire three-dimensional scans of whole tumor resection specimens using intraoral scanners (Figure 3). Based on these three-dimensional datasets, we propose a workflow for a digitized, specimen-based frozen section procedure: Commercially available intraoral scanners include software, which provides the option to modify the dataset by adding colored or three-dimensional structures and to share the datasets with other specialists involved in the patient’s treatment. In the proposed workflow, these options, usually used for the manufacturing of dental restorations, are used as a communication tool between the surgeon and the pathologist. In a first step, the surgeon marks locations that are considered to be high-risk areas in the 3D dataset. Next, the dataset and surgical cancer specimen are sent to the pathologist, who performs a specimen-based frozen section analysis. Margins of the specimen determined cancer positive by histopathological analysis are then marked in the 3D dataset by incorporation of three-dimensional virtual markers. The enhanced dataset is subsequently converted into the STL-format, facilitating its incorporation into the patient’s virtual dataset. This provides the surgeon with the information required to precisely target and resect any remaining cancer.

## Three-dimensional visualization of the tumor resection surface

### Disciplines involved: oral and maxillofacial surgery, ENT, radiotherapy

Besides of primary surgical resection of the tumor ENT (including tumor-free safety margins), adjuvant radiation therapy (RT) is an important component of the multidisciplinary treatment approach in head and neck cancer patients. According to the respective guidelines, adjuvant RT is indicated in case of advanced tumor, close or positive resection margins, and cases of cervical lymph node, vascular and/or perineural involvement [30].

High precision RT approaches, such as intensity modulated radiation therapy (IMRT), facilitate targeted delivery of the radiation dose to the tumor bed, which can contribute to an increased local control rate. At the same time, the radiation dose delivered to surrounding healthy native and reconstructed tissue can be decreased, reducing the effects of radiotherapy toxicity [3]. This can be considered to be of particular importance in patients with free flap reconstruction performed after tumor resection, as the effects RT has on the vascular system might negatively impact the outcome in these patients [31, 32].

Traditionally, radiation treatment planning is based on combined information from (pre- and postoperative) radiological imaging, pathology reports and operative notes [33]. A key requirement in the context of IMRT is precise delineation of the interface between the tumor resection margin and native/reconstructed tissue in postoperative imaging to prevent the risk of “marginal miss,” i.e., underdosing in the area of the target volume and to avoid the risk of overdosing in the area of healthy tissue at the same time [34]. However, in case of reconstruction of large resection defects using vascularized free flaps, which is often necessary in oral cancer patients, similar contrast values between native and reconstructed tissue impair subsequent delineation of the tumor resection surface [35, 36]. Intraoperative virtual marking of the resection surface can facilitate the identification of this critical structure in postoperative imaging. However, while confirmed to be accurate in the area of the craniofacial skeleton, CAS alone is insufficient in marking soft tissue resection margins distant from bony structures due to the effects of gravity and postoperative tissue rearrangement [37]. To overcome this issue, a technique combining 3D imaging, intraoperative navigation, and intraoperative incorporation of radiopaque physical markers (titanium ligature clips) has been described [19]. This technique allows for the detection of shifts of soft tissue in postoperative imaging, ultimately facilitating precise three-dimensional virtual reconstruction of the whole resection margin/surface, guided by the virtual and radiopaque physical markers in the fused pre- and postoperative imaging. This information can be made available to the radiation oncologist via an interdisciplinary server to facilitate precise planning of postoperative IMRT. In a clinical study, it could be demonstrated that this approach facilitates a significant reduction in the radiation dose administered to the graft with respect to the central part of the free flap while maintaining the boost dose to the planning target volume, including the tumor bed [36].

While metal-based fiducial markers like titanium clips are used in clinical routine (mainly in breast cancer surgery) since the early 1990s, injectable liquid fiducial markers represent an approach that has emerged more recently. As defined by Habermehl et al., the key characteristics for fiducial markers are (1) importance of visibility, (2) absence of artefacts, (3) easy application, and (4) sufficient immovability [38]. Considering these requirements, injectable liquid fiducial markers might be an advantageous alternative to titanium clips [39], [40], [41], [42], [43], [44].

**Figure 4:**
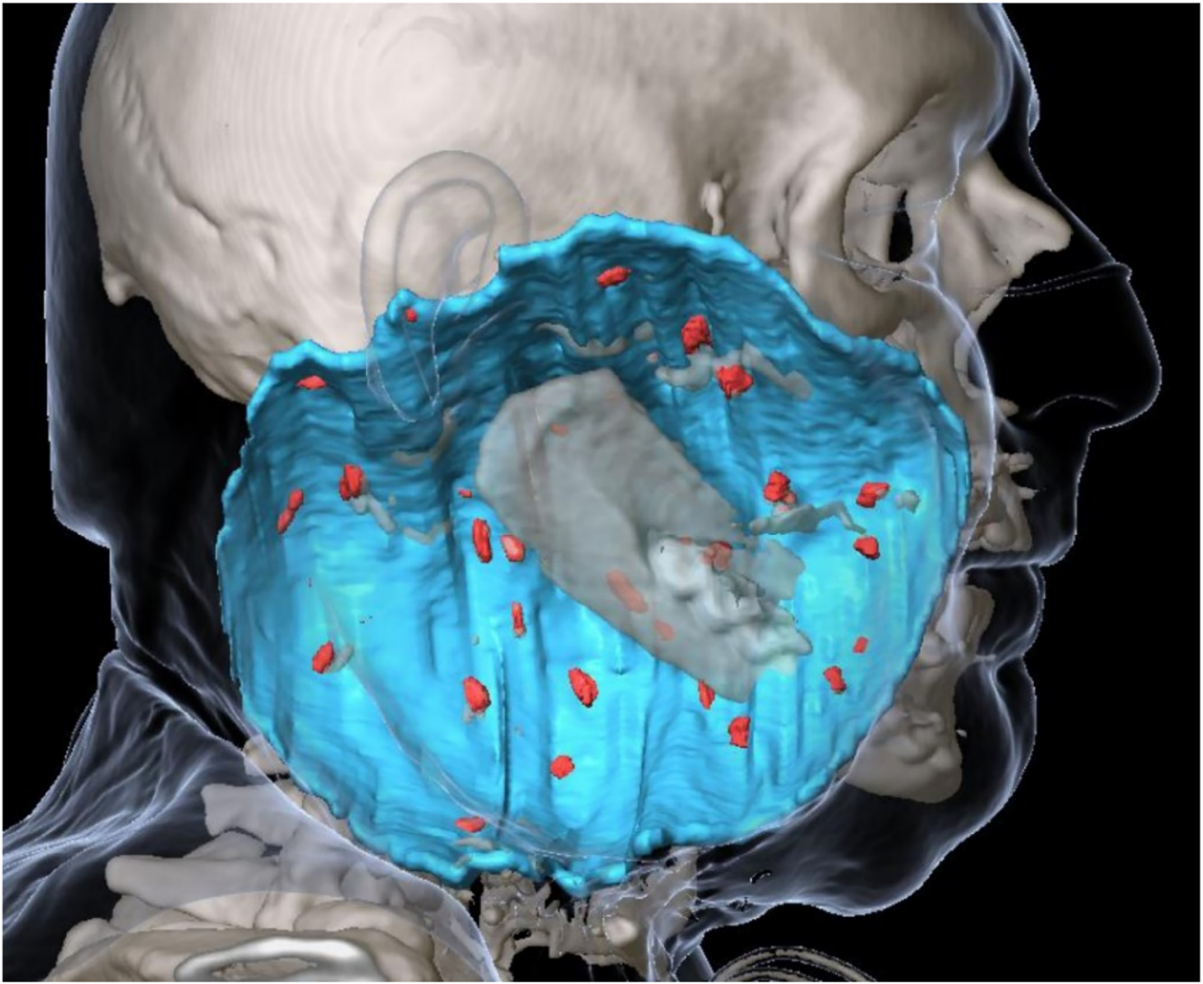
Virtual reconstruction of the tumor resection surface (blue) based on markers (red) created by intraoperative injection of a liquid fiducial marker in a patient with resection of an extensive tumor of the parotid gland and subsequent reconstruction with a scapula and latissimus dorsi flap.

The results obtained in a preclinical study on a novel CE-approved injectable liquid fiducial marker composed of sucrose acetate isobutyrate (SAIB) and iodinated SAIB (x-SAIB) suggest that this might provide a fast and reliable way for intraoperative postresectional creation of a high number of radiopaque markers at the soft tissue resection surface in head and neck cancer patients [45]. As very low volumes of this marker (10 μL) are sufficient to create fiducial markers with visibility on CT and CBCT comparable to the visibility of titanium ligature clips, a high number of markers can be placed, resulting in low distances to be interpolated between markers. This facilitates precise virtual reconstruction of the tumor resection surface in postoperative imaging (Figure 4). Moreover, markers that are delineable by their visible size in postoperative imaging can be created by simply altering the injected volume. This provides the option to mark, e.g., high-risk regions by using a volume different from the volume used for marking the resection surface in general. This way, these specific regions can be delineated in postoperative imaging and the respective information can be applied for RT planning. Apart from facilitating precise RT planning, by fusing medical imaging acquired during patient follow-up, the markers might also help in identifying three-dimensional structural changes of the tumor resection surface, potentially suggestive for recurrent cancer.

## Virtual 3D tumor reconstruction

### Disciplines involved: oral and maxillofacial surgery, ENT, pathology, radiology

Currently, information on the tumor and on ENT specific characteristics of the tumor obtained postoperatively is usually stored and shared using written reports. While this approach is sufficient for a number of clinical routine procedures, it makes it impossible to precisely store and share three-dimensional spatial information.

Approaches for three-dimensional virtual reconstruction of the tumor specimen have been described for different tumor entities [46], [47], [48]. While differing in some details, all of these approaches are based on a common workflow: In a first step, the resected specimen is placed in a cast and fixated in its position by filling the cast with agarose. Fiducial markers are placed in the cast and/or the specimen for orientation purposes. Subsequently, an ex vivo CT of the specimen is acquired, which is used to minimize registration errors during further processing. The cast containing the specimen is cut in 3–4 mm step sections in the same angle as the ex vivo CT slices. These macroscopic slices are digitized using a digital camera or scanner. For each macroscopic slice, a 4 μm section is obtained and stained for pathohistological analysis. These slices are subsequently digitized and co-registered with the corresponding macroscopic slices. Using the radiopaque fiducial markers as an orientation, these data can be co-registered to the ex vivo CT scan and transferred into the patient’s preoperative *in vivo* imaging.

To date, this technique has mainly been used to evaluate different imaging modalities/protocols for precise preoperative delineation of the tumor volume in the context of RT planning. Information on the tumor volume plays an important role in head and neck cancer RT and surgical planning. Novel imaging protocols for precise preoperative delineation of the tumor volume keep emerging in this area [49, 50] and three-dimensionally reconstructed tumor volumes could make a useful contribution to their evaluation and validation. Moreover, a lot of progress has been made in the past years regarding approaches for automated delineation of the tumor volume, which are based on convolutional neural networks (CNNs) [51]. In this context, three-dimensionally reconstructed tumor volumes could help in training and validating these CNN and thus contribute to the further development of this technique.

Another major field of application for three-dimensional tumor reconstruction might be the documentation and visualization of areas of the tumor with expression of certain molecular markers (“biodigitization”) by incorporation of (immunohistochemical) pathohistological findings into these datasets. The role of numerous molecular alterations in carcinogenesis, tumor progression, and recurrence is well documented. Obtaining three-dimensionally visualized spatial information on intratumoral distribution of these molecular pathologic processes provides the option of matching this information with imaging and pathologic information obtained during patient follow-up and in case of (locoregional) recurrence. This could help in improving the understanding of the role of these molecular processes in head and neck cancer. In this context, an area of particular interest might be the surgical margin, as a number of studies have reported that microscopically clear margins can still contain molecular alterations, resulting in an increased risk for locoregional recurrence [52].

## Conclusions

In the past decades, computer technology has transformed surgery in various ways and novel innovations keep emerging. In this context, data-driven approaches are expected to pave the way for major transformations in various aspects of surgical care [53].

Regarding cancers of the head and neck, promising findings are reported for technology-based approaches in key treatment components like preoperative planning, frozen section analysis, or adjuvant radiation therapy. Three-dimensional visualization, storage, and network-based interdisciplinary exchange of patient data provide the basis for the implementation of strategies that take full advantage of existing and upcoming technology-based therapy approaches in this field. This allows for refinements of established treatment components like frozen section analysis and postoperative radiation therapy as well as for novel approaches like visualization and storage of the three-dimensional intratumoral distribution of molecular markers and thus has the potential to make a major contribution to improving the outcome in head and neck cancer patients.

## Supplementary Material

Supplementary Material
