# Supplementary material for: Virtual tumor mapping and margin control with 3-D planning and navigation
Source: Innov Surg Sci. 2024 Apr 25;9(1):17–24. doi: 10.1515/iss-2021-0009 (PMC11138405; doi:10.1515/iss-2021-0009)
Supplement: Supplementary file 1 — Supplementary Material [file j_iss-2021-0009_suppl_001.pdf]

Reviewer Assessment

P. Poxleitner et al.: Virtual tumor mapping and margin control with 3-D planning and navigation

Reviewers' Comments to Original Submission

Reviewer 1: Werner, Jens

Date received: 07-Dec-2021
Reviewer recommendation: **Return to author for minor modifications**
Reviewer overall scoring: **High**

Comments to author:
This is a nice review on computer technology based treatment approaches for head&neck Cancers. The subchapters are well chosen aand written. SInce the article is directed towards beginners in the field, some additional examples should be included in the article.
.

Reviewer 2: Gellrich, Nils-Claudius

Date received: 28-Jul-2022
Reviewer recommendation: **Return to author for minor modifications**
Reviewer overall scoring: **Excellent**

Comments to author:
Minor corrections only. See my comments in the text

Comments by the Editorial Office to the Editor-in-Chief Decision

All reviewer comments were addressed adequately and the manuscript should be published in its present stage.
